# Genetic basis of transcriptome differences between the founder strains of the rat HXB/BXH recombinant inbred panel

**DOI:** 10.1186/gb-2012-13-4-r31

**Published:** 2012-04-27

**Authors:** Marieke Simonis, Santosh S Atanur, Sam Linsen, Victor Guryev, Frans-Paul Ruzius, Laurence Game, Nico Lansu, Ewart de Bruijn, Sebastiaan van Heesch, Steven JM Jones, Michal Pravenec, Tim J Aitman, Edwin Cuppen

**Affiliations:** 1 Genome Biology Group, Hubrecht Institute, Uppsalalaan 8, 3584 CT Utrecht, The Netherlands; 2 Physiological Genomic and Medicine Group, Medical Research Councils Clinical Sciences Centre, Faculty of Medicine, Imperial College London, Hammersmith Hospital, London W12 ONN, UK; 3 Genomics Laboratory, MRC Clinical Sciences Centre, Faculty of Medicine, Imperial College London, Hammersmith Hospital, London W12 ONN, UK; 4 Genome Sciences Centre, BC Cancer Agency, Suite 100 570 West 7th Avenue, Vancouver, British Columbia, Canada V5Z 4S6; 5 Institute of Physiology, Academy of Sciences of the Czech Republic, Videnska 1083, 14220 Prague 4, Czech Republic

## Abstract

**Background:**

With the advent of next generation sequencing it has become possible to detect genomic variation on a large scale. However, predicting which genomic variants are damaging to gene function remains a challenge, as knowledge of the effects of genomic variation on gene expression is still limited. Recombinant inbred panels are powerful tools to study the *cis *and *trans *effects of genetic variation on molecular phenotypes such as gene expression.

**Results:**

We generated a comprehensive inventory of genomic differences between the two founder strains of the rat HXB/BXH recombinant inbred panel: SHR/OlaIpcv and BN-*Lx*/Cub. We identified 3.2 million single nucleotide variants, 425,924 small insertions and deletions, 907 copy number changes and 1,094 large structural genetic variants. RNA-sequencing analyses on liver tissue of the two strains identified 532 differentially expressed genes and 40 alterations in transcript structure. We identified both coding and non-coding variants that correlate with differential expression and alternative splicing. Furthermore, structural variants, in particular gene duplications, show a strong correlation with transcriptome alterations.

**Conclusions:**

We show that the panel is a good model for assessing the genetic basis of phenotypic heterogeneity and for providing insights into possible underlying molecular mechanisms. Our results reveal a high diversity and complexity underlying quantitative and qualitative transcriptional differences.

## Background

Thanks to technological developments in next generation DNA sequencing the amounts of genetic variants that are identified in healthy and diseased individuals are growing rapidly. This has increased the potential of personal genomics: the sequencing of complete genomes with the intention to guide prevention or treatment strategies. For personal genomics to become a reality, however, it is important not only to systematically make inventories of genome-wide genetic variation, but also to know which genetic alterations can affect gene function [1,2]. Studying the effects of genomic variation in human populations is difficult, due to the large number of variants in every individual. Moreover, alleles can be heterozygous and homozygous with both recessive and dominant effects.

Here we have systematically studied the effects of genomic variants using two inbred rat strains, the spontaneously hypertensive rat (SHR/OlaIpcv) and a Brown Norway-derived strain, BN-*Lx*/Cub [3,4]. We refer to the strains as SHR and BN-*Lx *in this paper. The advantage of using inbred animals for genetic studies is that essentially all positions in the genome are homozygous. In addition, the strains form a renewable biological source, which makes it possible to analyze biological replicates and to do extensive validation studies. The SHR and BN-*Lx *strains are a particularly valuable system for studying the phenotypic effects of genetic variation, because they are the founders of a recombinant inbred (RI) panel, which is called HXB/BXH.

RI panels are created by crossing two inbred lines. Through inbreeding of the offspring new inbred lines are established that are mosaics of the two founder strains. The HXB/BXH RI panel consists of 30 lines that have been inbred for currently >80 generations. Extensive phenotyping has been performed at the physiological, behavioral and molecular levels (reviewed in [5]). Genetic maps were generated and quantitative trait loci (QTLs) have been identified through linkage analyses in the panel (reviewed in [5]). By analyzing gene expression levels in the RI panel, thousands of local (*cis*) and remote (*trans*) genetic loci that affect transcription, called expression QTLs or eQTLs, have been identified [6] and transcriptional networks have been dissected [7]. Due to the high level of synteny between rat and human, results obtained in the rat have been successfully extrapolated to human (reviewed in [8]). For example, a QTL study in a rat RI panel has recently led to the identification of the *ENDOG *gene as an important regulator of cardiac hypertrophy. The homologous gene in human was shown to be involved in the same biological processes as the rat gene [9].

To use the HXB/BXH RI panel for functional genetic analyses, a comprehensive inventory of the genomic differences between the founder strains is required. Moreover, such a resource will be essential to identify causal genes and variants that underlie physiological phenotypes in QTL regions. The genome of SHR was sequenced previously to 10× coverage [10]. This was shown to be instrumental for the identification of transcriptional regulators [7].

Here we re-sequenced the genome of BN-*Lx*. In addition, we updated the sequence of SHR by increasing the sequencing depth and employing novel analysis methods [10]. An extensive spectrum of genomic variants was characterized; single nucleotide variants (SNVs), insertions and deletions smaller that 10 bp (indels), copy number variants (CNVs) and structural genetic variants (SVs). SVs include deletions insertions tandem duplications and inversions.

We also performed RNA sequencing on liver samples of SHR and BN-*Lx *and identified both qualitative and quantitative differences in the transcriptomes. Analyses on the effects of the different genomic variants show that SVs, and in particular gene duplications, strongly correlate with quantitative transcriptomic output. Furthermore, combining genetic variation inventories with RNA-seq analyses provides clues on the mechanism of qualitative transcriptional alterations.

## Results

### Genome sequencing data

**Figure 1:**
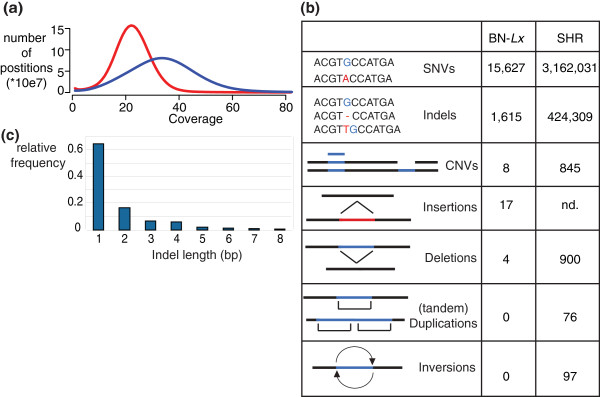
**Full genome sequencing identifies broad spectrum of genetic variants**. **(a) **Coverage profiles of SHR and BN-*Lx*. **(b) **The amount of different types of genomic variants detected in BN-*Lx *and SHR. **(c) **The distribution of sizes of indels that were detected. Nd, not determined.

A summary of the next generation sequencing (NGS) data produced in this study is shown in Additional file 1. We previously sequenced the SHR genome with median coverage of 10× [10]. By expanding the NGS data set of SHR the median coverage of this genome was increased to 23× (Figure 1a). For the analyses of the BN-*Lx *genome we produced three independent fragment libraries and a long mate-pair library. Combined this resulted in 33× median base coverage of this genome (Figure 1a). The BN/NHsdMcwi strain has been used to create the reference genome of the rat [11]. We refer to this strain as the reference BN. This strain was sequenced by traditional capillary sequencing and has not been covered to the same depth as we generated here for BN-*Lx *and SHR. Lack of coverage in the reference may contribute to false discovery of genomic variants in BN-*Lx *and SHR. To prevent false discoveries, we used novel whole genome NGS data that were generated from material from the same animal (named Eve) that was used to create the BN reference sequence. We used these data from the BN reference with 32× NGS coverage to filter for likely reference genome errors.

### Small genomic variants

We used two variant calling algorithms for SNV detection, which were employed on the SHR, BN-*Lx *and BN reference data. For each SNV position found in either BN-*Lx *or SHR we checked that the SNV was not found in the reference BN sample. We took the SNVs found by both callers to come to the final SNV set reported here (Additional file 2 contains a list of all SNVs in SHR; Additional file 3 contains a list of all SNVs identified in BN-*Lx*). The results of both SNV callers can be viewed in separate tracks in the Rat Genome Database [12].

We detected 15,627 homozygous SNVs unique to BN-*Lx *and 3,162,031 unique to SHR (Figure 1b) as compared, respectively, to the BN reference, adding up to a total of 3,177,658 SNVs within the RI panel. Capillary sequencing confirmed 70 of the 70 randomly selected homozygous SNVs (100%; Additional file 4). Although inbred strains are expected to be homozygous at each position, we also found 17,544 heterozygous positions specific for SHR and 225 specific for BN-*Lx*. Possible sources of heterozygous SNVs include recent *de novo *mutations, sequencing noise, incomplete inbreeding and diverged duplications. The latter two mechanisms are expected to result in clustering of heterozygous positions, whereas noise and *de novo *mutations would appear randomly. We analyzed the genome of SHR in 100 kb windows and found that the distribution of heterozygous SNVs is far from random; 84% of the heterozygous SNVs locate to windows with more than three SNVS. Of simulated randomly positioned SNVs, only 2.6% are located in such SNV-dense windows. Furthermore, 91 of the 148 genomic regions with the highest density of heterozygous SNVs (top 1%) were found to overlap with duplications, indicating that diverged duplications are a major source of heterozygous SNVs.

The homozygous SNVs between BN-*Lx *and the BN reference genome are also not distributed randomly across the genome. There are eight regions with a high SNV density, with a combined length of 51 Mb, together holding 97% of the SNVs (Figure S1A in Additional file 5). One of these regions, on chromosome 8, was expected to have a high density of SNVs because BN-*Lx *is known to be congenic for this region, which contains the *Lx *locus [4]. The discovery of the other seven regions demonstrates that there are more sub-chromosomal differences between BN-*Lx *and the reference than was expected based on the breeding history. These differential regions could be remnants of the original cross with the PD strain that created the congenic strain that was the founder for BN-*Lx*. To investigate this possibility, we analyzed a subset of the SNVs in other rat strains. Saar *et al*. [13] previously characterized 127 SNV positions in the 7 regions that differ between BN-*Lx *and the reference genome by high-throughput genotyping in 167 strains and substrains. Nine BN substrains from different institutes were included in this study. We found that a subset of the BN substrains has the same genotype in the seven polymorphic regions as BN-*Lx *(Figure S1B in Additional file 5). This shows that variants likely reflect the background of the BN strain that was used as a founder for generating the BN-*Lx *strain rather than remnants of the PD strain.

We did not find genomic regions with increased SNV density in SHR. We detected 220,332 additional SNVs that were not found in the analyses based on the 10× coverage of the SHR genome [10]. We also found 76,019 positions that were previously called homozygous SNV to be also non-reference in BN (6,077 positions), BN-*Lx *(28,751 positions) or both (41,191 positions), indicating that these most likely reflect errors in the reference genome assembly.

Next, we used the overlap between the two independent analysis tools to reliably call indels in the two rat strains. We identified 1,615 indels specific for BN-*Lx *and 424,309 specific for SHR (Figure 1b; Additional files 6 and 7 for SHR and BN-*Lx*, respectively). We analyzed 57 indels with capillary sequencing and confirmed all 57 (100%; Additional file 8). We called indels ranging from one to ten base pairs; 36% of the indels are larger than 1 bp (Figure 1c) and a large proportion (51%) of the indels are changes in the length of homopolymer stretches longer than 3 bp (82% of these involve A or T stretches).

Fifty percent of the SHR indels were not found in the previous analysis, and 27,416 of the 343,243 indels detected in the previous analyses were found to be not specific to SHR, of which 19,138 were found in both BN-*Lx *and the reference strain. These differences are not only the result of a higher base coverage, but are also due to a change in analysis methods. The previous report was based on a combination of MAQ and BLAT alignments [10] and here we used two independent indel calling algorithms. Moreover, the previous set was not corrected for reference errors.

As was seen for the SNVs, the indels that are seen in BN-*Lx *compared to the BN reference are found clustered in the genome; 93% of the BN-*Lx *indels are found in the eight regions that are different between BN-*Lx *and the reference.

### Structural variation

We detected SVs by two independent methods: based on raw read coverage and by long mate-pair (LMP) analyses.

NGS coverage differences between either SHR or BN-*Lx *and the reference BN can be used to detect changes in copy number. Using a dynamic window binning approach to identify genomic segments with differential NGS read coverage (DWAC-Seq; VG, manuscript in preparation), we identified four duplications in BN-*Lx *and four deletions compared to the BN reference, including a known 3 kb deletion on chromosome 8 [14]. In SHR we identified 384 duplications and 461 deletions (Figure 1b). The duplications ranged from 1,028 bp to 270 kb covering 7.3 Mb in total, and the deletions ranged from 1,036 bp to 2.3 Mb covering 11.7 Mb in total.

Some SVs are not detectable based on sequence coverage due to the repetitive nature of the sequence content or because they are copy neutral. Therefore, we analyzed LMP data for SHR and BN-*Lx*. In brief, LMP analysis involves DNA fragmentation, selection of fragments of a specific size range and sequencing of the ends of these selected fragments. The sequenced ends are aligned to the reference sequence and are expected to align at a distance from each other that matches the selected fragment size range. Mate-pairs that map at a different distance or in an unexpected orientation pinpoint changes in genome structure, which can be deletions, tandem duplications, insertions, inversions or translocations [15]. The SHR LMP library had a median insert size of 2 kb [10] and as a control we produced a LMP library of the reference BN with the same insert size. We found 76 tandem duplications, 97 inversions and 900 deletions specific for SHR. The mate-pair library of BN-*Lx *has a median insert size of 5.5 kb. In BN-*Lx *we identified 4 deletions and the large insert size of the BN-*Lx *mate-pair library allowed the identification of 17 insertions (Figure 1b).

The analyses on NGS coverage and LMP libraries combined results in a comprehensive list of SVs in SHR and BN-*Lx *versus the reference BN (Additional file 9).

### Transcriptome variations in liver

**Figure 2:**
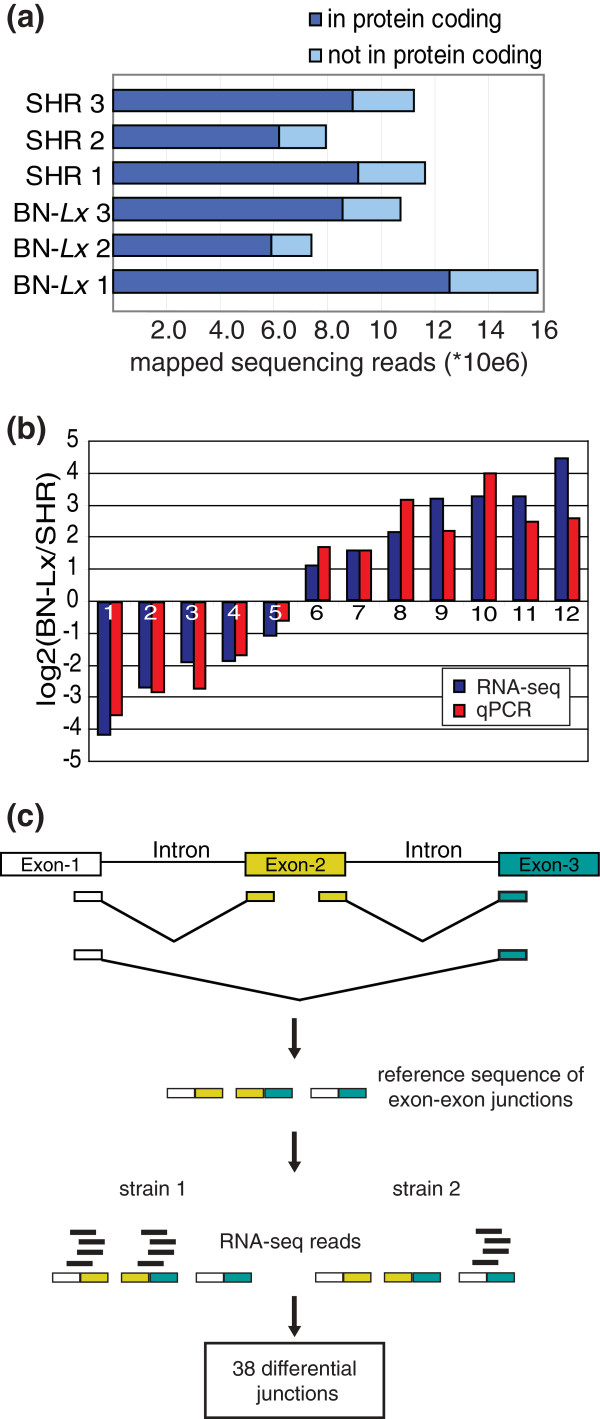
**The transcriptomes of SHR and BN-*Lx *liver have differences in both expression levels and splicing events**. **(a) **The amount of sequencing reads that could be mapped to the genome per RNA-seq library. Three animals were analyzed for each strain. **(b) **Twelve genes found to be differentially expressed between BN-*Lx *and SHR with RNA-seq were analyzed with qPCR. **(c) **Schematic representation of alternative splicing analyses. To analyze splicing events a novel reference was created by combining the sequences of the annotated exon ends. RNA-seq reads were mapped to this reference and junctions used differentially by BN-*Lx *and SHR were determined.

To investigate the effects of the genomic variation on transcriptome characteristics, we performed RNA sequencing on liver samples of SHR and BN-*Lx*^1^. We sequenced the mRNA of three animals of each strain to control for inter-animal diversity. By polyA and 5'-cap selection we obtained the full length mRNA products from the pool of total RNA. Strand information was preserved in the library preparation procedure. We obtained over 7.4 million mapped reads for each animal and 78 to 80% of the reads mapped to coding sequences (Figure 2a).

Using normalized read counts per gene as a measure for gene expression and taking intra-strain variation into account, we identified 532 differentially expressed genes with a minimum of a two-fold difference between BN-*Lx *and SHR (false discovery rate (FDR) <5%). We randomly selected 12 differentially expressed genes that differed between 2 and 21 fold and analyzed them by quantitative PCR (qPCR). Expression of all 12 genes was confirmed to be significantly different between SHR and BN-*Lx *in the qPCR experiments (*P*-values 9.8 × 10E-09 to 0.04, one sided two sample *t*-test, assuming equal variance) and the fold changes showed a median 1.6-fold difference between the two methods (Figure 2b). The qPCR experiments thus confirm our RNA-seq analyses.

We also investigated the differences in splicing events between the two rat strains (Figure 2c). For this purpose we created a reference transcriptome consisting of all combinations of annotated exons. We mapped the RNA-seq data to this transcriptome reference and quantified sequencing reads that mapped across exon-exon junctions. Because the goal was to relate splicing differences to genetic variation, rather than to identify all splicing variants in the transcriptome, we selected the exon-exon junctions with the largest differences between the strains. We found 38 junctions with large differences in coverage between SHR and BN-*Lx *in 36 genes; 27 of the junctions are annotated and 11 are new combinations within a gene (Additional file 10).

Thus, there are both quantitative and qualitative differences between the transcriptomes of BN-*Lx *and SHR in liver tissue.

### Genomic variation in coding parts of differentially expressed genes

Next we combined genomic and RNA-seq data to gain insight into potential direct effects of different types of genetic variants on the transcriptional output.

Combining the genomic sequence data from SHR and BN-*Lx*, we found 25,271 SNVs in coding positions, 8,830 non-synonymous coding, 129 in essential splice sites, 99 SNVs that cause a stop codon gain and 8 SNVs that cause a stop codon loss (Additional file 11 lists the affected genes and if they were expressed and differentially expressed in liver tissue).

There are 402 indels located in coding sequence, including 26 in essential splice sites and 260 indels that cause a frameshift (Additional file 12 lists the affected genes and if they were expressed and differentially expressed in liver tissue). Another 1,394 and 119 indels are located in 3' and 5' UTRs, respectively. Together, 1% of the SNVs and indels are located in coding regions or splice sites, and 489 genes are affected by essential splice site, stop or frameshift variants.

Of the SVs (which include CNVs and structural variants called in the LMP analyses), 6% contain at least parts of coding sequences, together affecting 108 genes (Additional file 13 lists the affected genes and if they were expressed and differentially expressed in liver tissue).

**Figure 3:**
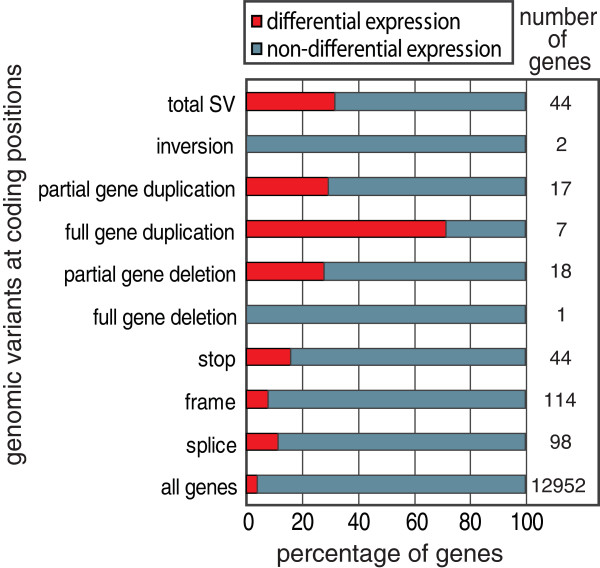
**Different types of genomic variants have different predictive value**. For each type of genomic variant the genes that contained such a variant were analyzed. The amount of expressed genes that overlapped each type of variant type is given in the right column. The proportion of these genes that is differentially expressed between SHR and BN-*Lx *is represented by the red bars.

We investigated which types of genomic variants are most likely to alter gene expression - in other words, which have the highest predictive values. We focused on the expressed genes for each variant and determined the fraction of genes that showed differential expression levels between BN-*Lx *and SHR (Figure 3). Deletions and duplications are often called by both CNV and LMP analyses. We therefore combined these sets to one list of genes affected by duplications and one list of genes affected by deletions (relative contribution of both analyses methods is given in Additional file 13).

We found that full gene duplications have the highest predictive value (Figure 3). The fraction of genes that is differentially expressed is relatively low in the gene set carrying SNVs. To gain insight into these differences, we studied these effects of the different types of variants in more detail.

### Small genomic variants versus differential expression levels

Novel stop codons can lead to degradation of the mRNA through nonsense-mediated decay. Stop codons can be introduced by SNVs and by frameshifts, which can result from indels or splice site changes; 193 genes carrying one of these types of variants are expressed in liver, and only 19 of these are differentially expressed between SHR and BN-*Lx *(Figure 3).

To verify that the lack of effect of stop mutations on transcript levels is not due to false positive genomic variants, we analyzed a subset with capillary sequencing. We sequenced 17 SNVs that were predicted to create a stop codon and were located in a gene that is expressed in liver. All 17 positions were true SNVs (Additional file 14). This demonstrates that the lack of change in expression is not due to false variant predictions.

An alternative hypothesis is that the functional predictions are invalid. Visual inspection of the RNA-seq data revealed that many of the stop variants in the unaffected genes are located in exons that are not included in the transcript in liver. Functional predictions can be improved when data on alternative transcript usage become more widely available. In addition, we found many examples where inaccurate annotation results in erroneous reading frame depictions and, as a result, in false stop codon predictions. Thus, the limited effect of predicted stop mutations on transcript levels is not due to false variant detection, but rather the result of functional predictions that are hampered by incomplete and inaccurate gene annotation.

If functional predictions are improved, the total the amount of SNVs and indels that are predicted to change stop codons will likely decrease. The fraction of the stop variants that has an effect on transcript level would then be expected to become higher. However, what can be estimated from the data presented here is that the total contribution of stop variants to transcriptome variation is low. Only 4% of the differentially expressed genes contain a stop variant. These analyses do not capture the full effects of novel stop codons. We have only investigated liver here and it is possible that the effects are larger in other tissues. Moreover, stop-codons could also result in truncated proteins, without affecting expression levels of the gene.

### SVs in coding sequences of differentially expressed genes

Intuitively, a full gene deletion is the type of SV most likely to change gene expression. However, gene deletions cannot contribute to variation in the transcriptome in liver tissue if the deleted genes are not active. Of the 13 genes that are predicted to be completely deleted, only one, *Uba2*, is expressed in liver and this gene does not show differential expression levels between BN-*Lx *and SHR. This specific deletion in SHR was only detected in the mate-pair analysis and was not found based on genomic coverage. This suggests the gene sequence is in the genome of SHR, but not at the same position as in the reference genome. It is possible that this gene translocated to a different part of the genome, but is still functional. Unfortunately, there were no mate-pairs with one end inside the deletion, so we could not determine the translocation site based on the LMP data. Alternatively, it is possible that the gene is not translocated and is really deleted, but that the deletion is missed in the coverage analyses.

**Figure 4:**
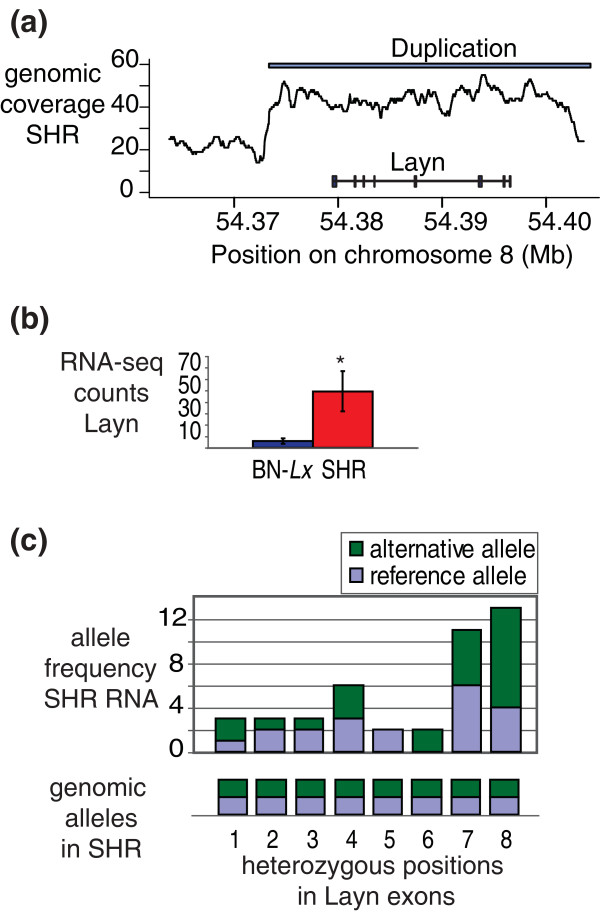
**Duplication of the *Layn *gene locus in SHR results in transcription from the original and the extra allele**. **(a) **Genomic coverage (averaged over 999 bp) in SHR at the *Mx2 *locus. The site of the duplication is indicated with the white bar. **(b) **Expression levels as measured by normalized RNA-seq read counts in the coding regions of the *MX2 *gene. The asterisk indicates significant difference (FDR <0.05). **(c) **Eight heterozygous SNVs were found in the exons of *Layn *in the genomic sequencing data of SHR. RNA-seq reads covering these positions were analyzed to investigate if they contained the reference or the alternative allele.

Gene duplications are strongly correlated with changes in expression. Five of the seven fully duplicated genes that are expressed in liver are differentially expressed. Three of the affected genes, *RT1-T24-1*, *RT1-CE1 *and *RT1-CE4*, reside in a highly polymorphic region on chromosome 20 coding for the *MHC1 *complex. This part of the genome shows a complex pattern of variation at the genomic and transcriptomic levels. We investigated the data for the other two differentially expressed genes found to be duplicated; *Mx2 *and *Layn*, in more detail (Figure 4 shows the analyses for *Layn*; Figure S2 in Additional file 5 shows the analyses for *Mx2*). Visual inspection of the coverage in the regions showed that both duplications cover only the differentially expressed gene and no other genes (Figure 4a; Figure S2A in Additional file 5). Both *Mx2 *and *Layn *show an increase in expression (Figure 4b; Figure S2B in Additional file 5). The elevated transcript levels could result from the fact that there are four copies of the genes per cell instead of two. Alternatively, the duplication of regulatory elements could result in up-regulation of the original two gene copies. To investigate these hypotheses, we analyzed the heterozygous SNVs called in the genome in the RNA-seq data. Both duplications contain heterozygous SNVs. These can arise from diversification of the sequence after duplication. Both the original and the duplicated sequence map to the same position in the reference genome and are detected as heterozygous SNVs. We focused on the heterozygous SNVs in the exons of the genes. All nine heterozygous genomic positions that are located in the *Mx2 *exons were also heterozygous in the RNA-seq data (Figure S2C in Additional file 5). In *Layn *6/8 heterozygous positions in the exons showed expression from both alleles (Figure 4c). In BN-*Lx*, which does not carry the duplications, all described positions were homozygous reference at the DNA and RNA levels. These data uniquely show that the transcripts of *Mx2 *and *Layn *are produced from two different alleles in SHR; the original locus and the extra copy.

**Figure 5:**
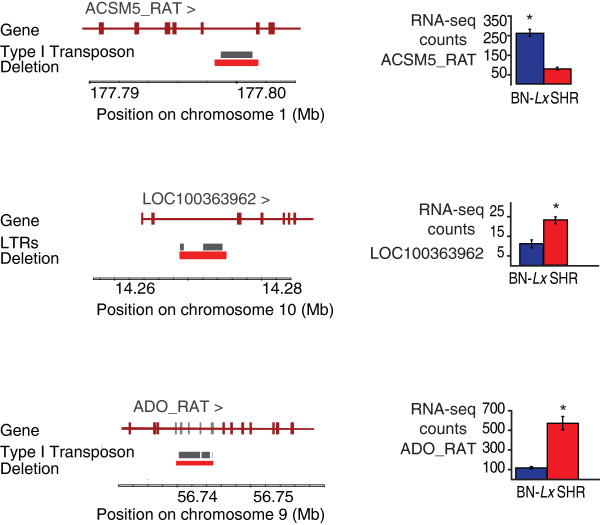
**SVs covering intronic repetitive elements correlate with changes in gene expression**. Three large intronic deletions (red bars) of repetitive elements (grey bars) correlate with a change in gene expression (right). Asterisks indicate significant change (FDR <0.05). Exons indicated in grey are not used in either strain, according to RNA-seq data.

Some duplications and deletions cover only parts of genes. There are 18 expressed genes with partial duplications, of which 5 are differentially expressed. Nineteen genes with predicted partial deletions are expressed in liver and five of these are differentially expressed. One of the genes that is partially deleted is located in the CD36 locus, which has a known functional deletion [16]. The deletion in *RT1-Bp *causes the loss of the last exon, described below. The other three partial deletions found in differentially expressed genes were on visual fine mapping and analyses of RNA-seq data all confined to intronic sequences. Characterization of the deleted sequences showed that they all contained >2,100 bp of a repetitive element: two were remnants of long interspersed elements (LINEs) and one was a long terminal repeat (LTR) element (Figure 5). This led us to examine other deletions in introns. Of the 100 deletions that contain >2 kb of repetitive sequence, 21 are located in introns. Nine of these genes are expressed in liver and four are differentially expressed. These examples suggest a possible effect on expression levels mediated by transposon-derived sequences in introns.

The above described findings on duplications and deletions demonstrate the potential of the HXB/BXH model system for the elucidation of SV-mediated changes in gene expression.

### Non-coding genomic variation and expression levels

An important part of the sequences that regulate gene expression is located outside the coding parts of genes. Therefore, we investigated the presence of genetic variation in the 5' and 3' UTRs and in the 5 kb region upstream of the transcriptional start sites. Of the expressed genes in liver tissue, 2979 have at least one SNV or indel in the 5' and or 3' UTR. Only 5% of these genes are differentially expressed. Of the total set of transcribed genes, 4% is differentially expressed. Thus, the presence of an SNV at a conserved position in a UTR does not make a gene much more likely to be differentially expressed. So what about the number of SNVs and indels in the regulatory regions? We investigated SNV and indel density in the differentially versus the non-differentially expressed gene set. We found that the differentially expressed genes contain, on average, 8.1 SNVs and 2.0 indels in the 5 kb upstream of the transcriptional start site. The number of small genomic variants is smaller in upstream regions of genes that are not differentially expressed; 6.6 SNVs and 1.7 indels on average. Although the absolute differences are small, they are highly significant (two tailed *t*-test, *P*-value 0.0003 and 0.005 for SNVs and indels, respectively). This is in accordance with the previous finding that *cis*-eQTL gene loci are enriched for SNVs and indels [10].

### Differential splicing and splice site mutations

**Figure 6:**
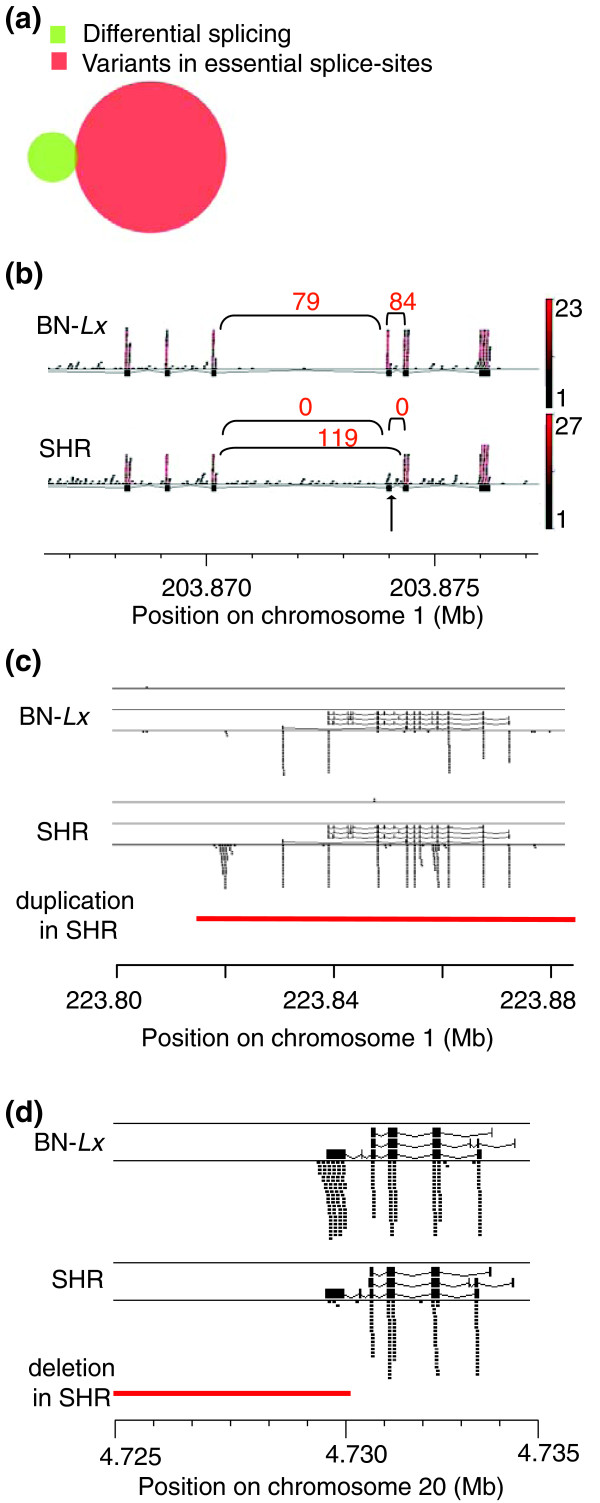
**Only a small part of the changes in transcript structures can be explained by underlying genomic variation**. **(a) **The overlap between splice junctions that are produced at different levels in SHR and BN-*Lx *and small genomic variants in essential splice sites. Only one of the splice junctions that is different in BN-*Lx *and SHR carries a variant in an essential splice site. **(b) **The single differential splice junction that overlaps with an SNV (arrow) results in exon skipping in *Slc22A18C*. The exons are indicated as black boxes. RNA-seq reads that mapped at this gene locus are shown as small horizontal bars. The red scale indicates the amount of times each read was found in the data set. The number of RNA-seq reads that mapped across the exon-exon junctions is represented in the numbers above the arcs. **(c) **Mapped RNA sequencing reads (small black bars) in *D3ZCV5_RAT *show a different transcript structure in BN-*Lx *versus SHR. The different transcripts in the current annotation are shown on top of the reads. The gene is duplicated in SHR (red bar) **(d) **A large deletion (red) in SHR covers the last exon of the *RT1-Bp *gene. The deleted exon shows little coverage in the RNA-seq data of SHR.

Next we analyzed the correlation between small genomic variants and splicing. There are 129 SNVs and 26 indels located in essential splice sites. The quantitative analyses of exon-exon junctions resulted in 36 genes with differential splicing. Only one of these contained a genomic variant, a SNV, directly in an essential splice site (Figure 6a). This sequence change resulted in the skipping of an exon in SHR (Figure 6b). The affected gene, *Slc22A18*, is imprinted in mouse and human and has been linked with tumorigenesis [17,18]. The exon loss does not lead to a frame-shift but results in a partial loss of a transmembrane domain, which could be deleterious to the function of the gene.

The lack of overlap between genomic variants and splicing variation is not simply because the genes with genomic variants in essential splice sites are not expressed in the investigated tissue. Sixty-nine of the genes with SNVs and 14 genes with indels in essential splice sites are expressed, but only 8 junctions have more than 2 reads mapped to them and none have more than 7 mapped reads. This suggests that, at least in liver, many transcripts are not spliced according to the current annotation.

### Alternative transcripts and genomic variants outside splice sites

The finding that the genes that are differentially spliced in BN-*Lx *and SHR do not contain genomic variants in essential splice sites suggests that the alternative splicing is caused by genomic variation at regulatory positions. Splicing can be regulated by splicing factors that bind to regulatory sites located in introns [19]. We therefore investigated the genomic variants in the introns of the affected sites. We selected the 27 splice junctions formed between exons that neighbor each other in the gene and, thus, cover only one intron. Of these 27 selected junctions, 17 had no genomic variant at all in the intron. To detect possible functional sites in the ten introns with genomic variants, we analyzed the phastCons9way conservation scores per variant base. This score represents the probability that a specific base is conserved, with 1.0 indicating most likely conserved [20]. All but one variant position had conservation scores below 0.1. The highest conservation was 0.26, but this SNV was not causative. In the intron with this SNV there is another SNV 10 bp before the acceptor site, which creates a new splice site and adds three amino acids to the protein, which was confirmed by sequencing the cDNA (Figure S3 in Additional data 5). The affected gene is the highly conserved *Fibrinogen alpha*.

Despite this one example, the vast majority of quantitative differences in splicing can not be linked with a genomic variant in the corresponding intron.

While investigating differentially expressed genes we found another source of alternative transcripts. Some of the genes that overlap SVs not only change the expression level of the annotated gene sequence, but also show novel alternative transcripts. For example, the uncharacterized gene *D3ZCV5_RAT*, which is duplicated in SHR, produces a different transcript than the locus in BN-*Lx *(Figure 6c). This gene shows differential expression, but was not included in the total analyses presented in Figure 3a because it is a novel gene without a Refseq ID. The RNA-seq data for this gene do not show that different exons are expressed in the duplicated locus in SHR (Figure 6c).

The highly polymorphic region of the MHC locus on chromosome 20 shows a mixture of different SVs, differences in expression levels and production of alternative transcripts (data not shown). There is one clear example of a partial deletion that resulted in the loss of an exon (Figure 6d), but more detailed functional analyses are necessary to elucidate the remainder of these exceptionally complex sites.

## Conclusions

We sequenced the SHR genome to high depth and produced a full genome analysis of BN-*Lx *to establish a comprehensive resource of nucleotide and structural genomic variants between these two founder strains of the HXB/BXH RI panel. We identified 3.2 million SNVs and 425,924 indels. Studies performed on three human individuals detected 3.4 [21], 3.2 [22] and 3.3 [23] milion SNVs and the numbers of indels detected in two of these studies were 170,202 [21] and 292,102 [22]. Thus, the number of SNVs and indels between the two investigated rat strains is highly comparable to the amounts found when human individuals are compared to the human reference genome [21-23].

In the model system used here the far majority of variation is homozygous because each strain is highly inbred. Environmental effects on expression are limited because the rats are kept in a controlled facility. Moreover, by analyzing transcriptomes of multiple animals of the same strain biological variation can be accounted for. Therefore, we could directly investigate the effect of genetic variation on quantitative and qualitative diversity in transcription *in vivo*.

It has been reported before that a large part of the variation in transcription levels can be assigned to CNVs [24,25] and that the level of contribution is related to the size of the CNV [26]. In contrast, recent studies on different inbred mouse strains have suggested a relatively small role for SVs on phenotypic and transcriptional variation. However, the strongest QTLs were enriched for effects of the variant type SV [27,28].

In line with these latter reports, we found that the total amount of genes affected by SVs is lower than that of the SNVs and indels (108 versus 489), but when SVs are involved, the effects are more likely to be prominent.

We have estimated which type of variants are the best predictors for differential expression by considering which proportion of the expressed genes carrying a specific type of mutation are differentially expressed. Using this criterion, duplications are the best predictors for differential expression. Expanding the analyses to more tissues and more rat strains can further generalize the predictive value of different types of genomic variants. Moreover, the predictive value of especially SNVs and indels can be different in organisms for which the gene annotation is more accurate - for example, in human. It should be noted that our assessments are aimed towards functional predictions based on whole genome analyses. The strong predictive value of stop and splice mutations as *de novo *events or as recessive alleles in congenital disease, for example, is not questioned here. However, our data do illustrate the importance of full spectrum analyses of genomic variants in (clinical) genetic studies.

Moreover, the data of specific genes are illustrative for the diversity of mechanisms by which SVs can change expression. By nucleotide analyses of RNA-seq data in gene amplifications we found examples that show gene amplification and subsequent diversification do not necessarily lead to pseudogenes, but can result in expression from both the original and the duplicated locus and can result in a novel transcript. Deletions of >2 kb of repetitive elements located in introns were found in genes with a change in level of transcription. It has been described that remnants of repetitive elements can form transcriptional start sites and binding sites for transcription factors. As it is also shown that the repetitive sequences are unstable sites in the genome and are thus likely to mutate, these sequences could be a major direct cause of transcriptional variation between individuals. Due to the limited size (3.0 to 6.4 kb) and the repetitive nature of the encompassed sequence, this type of deletion is largely missed in predictions based on NGS read coverage. These findings thus illustrate the importance of including mate-pair analyses in genetic studies.

Combining stop codon-related small genomic variants and the SVs, only 37 of the 532 differentially expressed genes contain a genomic variant with a predicted effect on gene expression. Equivalently, changes in transcript structure rarely overlap with genomic variants. This suggests that the major part of both qualitative and quantitative variation in the transcriptome is regulated by unknown regulatory elements or result from changes in transcriptional networks. Segregation analyses of the transcriptome in the RI panel can dissect these *cis *and *trans *regulatory factors, both for gene expression levels and splicing variation.

We have not evaluated the genetic content of the individual 30 RI lines. Some *de novo *mutations may have occurred in the 80 generations of breeding. Indeed, in a previous study evaluating CNV patterns in the founder lines and two of the RI lines, three novel CNVs were detected in the RI strains compared to 626 normally segregating CNVs [29]. Nevertheless, as lines are crossed without mixing from the F2 generation on, all novel variants will be private to a single line. Although such variants could affect biology, they do not affect the genotype-phenotype segregation pattern within the RI panel, which is the common type of use for this genetic system.

## Materials and methods

### Sequencing library preparation

Below we describe the detailed library preparation protocol. All enzymatic reactions were performed according to manufacturers' conditions unless indicated otherwise. We performed sample cleaning in between all enzymatic reactions. To keep the description below concise we only describe the cleanup once here: cleanup was performed using QIAquick cleanup kit (Qiagen, Venlo, The Netherlands) according to the manufacturer's instructions. Elution was performed by two incubations with 35 µl of EB and incubating at 20°C for 2 minutes.

For preparation of BN-*Lx *DNA fragment libraries genomic DNA from BN-*Lx *liver tissue was sheared using the Covaris sonicator (6 minutes, 6X 16 mm AFA fiber Tube, duty cycle 20%, intensity 5, cycles/burst 200 frequency sweeping). End repair was performed (creating blunt 5' phosphorylated ends) using the End-It kit (Epicentre, Madison, Wisconsin, USA). Double-stranded P1 and P2 adapters (Applied Biosystems, Carlsbad, California, USA) were ligated to 2 µg of sheared DNA using the Quick ligation kit (New England Biolabs, Ispwich, Suffolk, USA). Nick translation was performed on the ligation product using 10 U of DNA Polymerase I in Buffer 2 (New England Biolabs) and incubating at 16°C for 30 minutes. DNA fragments were size selected on 2.5% agarose gel, excising 125 to 175 bp fragments and using the MinElute Gel extraction kit (Qiagen). Libraries were amplified using Platinum Supermix (Invitrogen, Life Technologies Europe, Bleiswijk, The Netherlands). The number of PCR cycles used was determined in a trial titration PCR, such that the amplification was still exponential in the last cycle. Amplification conditions were: 95°C for 5 minutes × cycles of 95°C for 15 s, 62°C for 15 s and 70°C for 1 minute, followed by one cycle of 70°C for 5 minutes where 'X' is the number of cycles that was determined by the trial titration PCR.

The mate-pair library of BN-*Lx *was obtained by shearing liver genomic DNA using the Hydroshear (Digilab, Holliston, Massachusetts, USA). Sheared DNA (50 µg) was loaded on a 0.6% agarose gel and fragments of 5 to 7 kb were excised. Fragments were circularized incorporating an internal adapter (Applied Biosystems) using Quick ligase (New England Biolabs), applying it on 4 µg of DNA in a 900 ml reaction volume. Uncircularized DNA was removed using plasmid safe DNase (Epicentre). Nick translation was performed using 18 U of DNA Polymerase I (New Engalnd Biolabs) and incubating on ice for 13 minutes. The DNA circles were cut using T7 exonuclease (New Engaland Biolabs) and subsequently by S1 nuclease (New England Biolabs). End repair was performed using the End-It kit (Epicentre). The library was bound to 150 µl streptavidin coated dynal beads (Invitrogen) that were pre-washed with bovine serum albumin. P1 and P2 adapters were ligated to the DNA using Quick Ligase (New England Biolabs). The library was nick-translated using DNA Polymerase I in Buffer 2 (New England Biolabs) and amplified using Platinum Supermix (Invitrogen). Amplification conditions were: 95°C for 5 minutes followed by 15 cycles of 95°C for 15 s, 62°C for 15 s and 70°C for 1 minute, followed by 70°C for 5 minutes. The library was size-selected on a 2.5% agarose gel, excising the fragments between 225 and 250 bp in size and using the MinElute Gel extraction kit (Qiagen).

The construction of the LMP library for the reference BN was performed essentially the same as for BN-*Lx *except that the DNA fragments were excised between 2 and 4 kb.

In the preparation of RNA-seq libraries cleanup in between enzymatic reaction was performed by phenol-chloroform (pH 4.5) extractions. RNA-seq libraries were made starting with 50 µg total RNA from liver tissue per animal and selecting the poly-adenylated fraction using the micro polyA purist kit (Ambion, Life Technologies Europe, Bleiswijk, The Netherlands). Capped mRNA was selected using the eukaryotic mRNA only kit (Epicenter). Fragmentation of the RNA was performed by incubating in 20 µl 3mM MgCl, buffered in 10 mM Tris pH8 for 15 minutes at 95°C. SREK Adapter A (Applied Biosystems) was ligated to the fragmented sample in SREK hybridization solution (Applied Biosystems) and incubated for 5 minutes at 65°C. After chilling on ice, ligation was performed according to the SREK protocol. The samples were size-selected, excising 110 to 200 nucleotide fragments from a 10% denaturing polyacrylamide gel. Reverse transcription was performed using 200 U RT enzyme (Promega, Madison, Wisconsin, USA) and incubating at 37°C for 30 minutes. PCR amplification of the libraries was performed according to the SREK protocol followed by excision of 150 to 200 bp fragments from a 6% native polyacrylamide gel.

Illumina genome sequencing libraries for SHR were prepared using methods described previously [30]. Resulting libraries were sequenced on Illumina HiSeq-2000 according to the manufacturer's instructions.

### Next generation sequencing

To generate genome sequencing data for BN-*Lx*, one 36 bp fragment, two 50 bp fragments and one paired-end library were processed on AB/Solid version 2, AB/Solid version 3 and AB/Solid version 4, respectively. The mate-pair library of BN-*Lx *was sequenced on AB/Solid version 3 reading 40 bp from both the forward and the reverse tag. RNA-seq libraries were sequence on AB/Solid version 3, generating 50 bp reads. For SHR, 100 bp paired-end reads (2 × 100) were sequenced on Illumina HiSeq-2000 version 1.5 using libraries generated before [10] following the manufacturer's instructions.

The reference BN data used to filter our SNV and indel data set were produced on AB/Solid.

### SNV and indel calling

SNV and indels were called with two algorithms.

#### SNV and indel caller 1

All sequencing reads were mapped with Burrows-Wheeler Aligner (BWA-0.5.8c) (settings: -c -l 25 -k 2 -n 10) [31] to the BN/NHsdMcwi reference genome RGSC-3.4 [32]. SNVs and indels were called using an adapted version of Samtools pileup and custom perl scripts (available on request). Filtering of the SNVs and indels was done as follows: reads that map to multiple positions in the genome and reads that were likely to result from clonal expansion in one of the PCR steps in the sequencing protocol (reads with identical read start position and strand) were discarded. Alleles should be detected by at least three sequencing reads with a high mapping quality (>30) of the variant base. SNV positions where an indel was detected within 10 bp were discarded because indels give mapping artifacts that lead to false positive SNVs. The remaining potential variant positions were separated based on the percentage of the calls that are non-reference (pnr). Positions with a pnr smaller than 10 were called reference, between 25 and 75 were considered potential heterozygous, and above 75 were called homozygous SNVs. The potential heterozygous positions were filtered to have no more than three times the median coverage to avoid repeat related errors. Indels were called with a pnr of 40 and higher. This was done because indel positions are harder to map and are covered by more misaligned reads, which causes a drop in the percentage of reads that predict the indel.

Many false SNVs and indels will be systematic and detected in more than one rat strain. These false SNVs will include errors in the reference genome but also systematic noise, such as sequencing errors and mapping artifacts. We therefore used the three genome data sets to filter each other and selected SNV and indel positions found in only one strain that were called reference in the other two strains.

#### SNV and indel caller 2

Quality filtered Illumina paired-end as well as mate-pair reads for SHR were mapped to the BN/NHsdMcwi reference genome RGSC-3.4 [32] using the read alignment software Burrows-Wheeler Aligner (BWA-0.5.8c) [31] with all default parameters except for the read trimming parameter. Reads were trimmed if base quality value decreased to <20 at the base where read quality decreased to <20. SOLiD reads for the BN-*Lx *genome were mapped to the BN/NHsdMcwi reference genome using software BFAST [33] using default parameters and ten indexes.

Genomic variants (SNPs and short indels) were detected using the Genome Analysis Toolkit (GATK version 1.0.6001) [34,35]. Instead of calling variants independently in each strain/genome, variants were called in both the genomes simultaneously using the multisample variant calling functionality of GATK. This allows identification of genomic variants between two strains irrespective of the reference genome. Before calling variants, data were processed using various pre-processing steps including i) removal of clonal reads, ii) indel realignment, and iii) base quality recalibration. GATK variant quality score recalibration was used to filter out potential false positives. Based on SNP quality score, the top 30% SNPs were used as a training set to build the Gaussian mixture model. We used a cutoff such that 99% of the SNPs in the training set were included in the final SNP set.

#### SNV and indel overlap

The results of both SNV and indel callers were compared and SNVs and indels called uniquely in BN-*Lx *or SHR in both sets were used for downstream analyses described in the paper.

### CNVs and SVs

Sequence read coverage of BN-*Lx *and SHR were both compared to the BN reference using DWAC-seq (VG, unpublished data). This software tool employs a binary segmentation algorithm, using a dynamic window to compare coverage between two samples. A window size of 250 bp was applied. The analyses were done with both ambiguity 1 and ambiguity 10 (ambiguity = maximum number of hits per sequencing read that is allowed to include the read in the analyses) for BN-*Lx *and SHR. For each set deletions were detected with threshold of ratio sample/BN <0.4. For calling amplifications, ratio sample/BN>1.6 was required. The overlap between the sets called with ambiguity 1 and 10 was selected. CNVs that were detected in both strains were removed from the list.

Analyzing the mate-pair data, we scored deletions and tandem duplications found only in SHR and not in BN. We required at least three independent pairs to predict the variant. For inversions we required both breakpoints to be covered by at least one pair. For duplications we required the size to be larger than 3,100 bp. For deletions we analyzed the distribution of the distance between the mapping positions of pairs. We set the threshold at 1% of this distribution and required at least three of these pairs to overlap each other to call a deletion. In addition, the median insert size of all pairs spanning potential deletions was taken into account. Threshold was set at (median(SHR) - median(BN))/median(SHR) > 0.25.

### Differential expression

RNA sequencing NGS data were mapped using BWA (settings: -c -l 25 -k 2 -n 10). As a measure, the total number of reads mapping to the total coding sequence of a gene was used (gene annotation ensemble 56). Read numbers were normalized to the total number of reads mapping within coding sequences. A gene was called expressed if the average normalized read counts were >10 in at least one of the strains (SHR or BN-*Lx*). *t*-Test statistics were calculated for each gene. Genes were called differentially expressed if the difference was more than two-fold different with *P*-value <0.05. To correct for multiple testing effects, we swapped samples of strains and calculated *P*-values and fold change for each swap combination possible. In this total set we found, on average, 23 genes with *P *< 0.05 and a two-fold change. Of the 532 differentially expressed genes, 23 can be expected to be false. This resulted in a FDR of 23/532, thus an FDR <5%.

To verify mRNA level differences, qPCR was performed using BioRad's MyIQ™ iCycler (Veenendaal, The Netherlands). cDNA was generated from 1 μg of isolated mRNA using Ambion's RETROscript® Kit (#AM1710). All reactions were done in triplicates in a 96-well optical-well plate format. For each reaction 10 μl 2x iQ SYBR Green Supermix (BioRad #170-8880), 2 μl cDNA (1 ng/μl), 1.2 μl forward and reverse primer (0.8 μM final concentration) and 6.8 μl H2O were used. Reaction conditions were as follows: 3 minutes at 95°C followed by 40 cycles of 30 s at 95°C and 30 s at 59°C. 

Visualization of RNA-seq data was done in TagBrowser, available on request.

### Splicing

To detect changes in splicing we created a database of all exon sequence combinations (ensemble 56) of exons within 1 Mb of each other (gene annotation ensemble 56). RNA-seq reads were mapped to this database with BWA (settings: -c -l 25 -k 2 -n 10). Mapped reads were filtered to only map to one location and have less than five mismatches. The number of remaining reads were normalized to the total number of reads mapping to the genes and exons with mapping bias due to SNVs in the start or end of the exon being excluded. We then detected for junctions that were found more frequently in one of the two strains such that abs(A) >4 (at least 4 reads were in one of the strains), where A = log2(BN-*Lx*) - log2(SHR). 

### Linking genomic and transcriptomic variants

Functional prediction of SNVs and indels was performed using Variant effect predictor 2.0 [36]. To determine the overlap between SVs and genes, we selected known protein coding genes with a RefSeq ID. Conservations scores (phastCons9way) used were obtained from the UCSC Table Browser [37].

### Data availability

All genomic sequencing data are available in the Sequence Read Archive (SRA) [38]. BN-*Lx *genome data have accession number ERP001355. The new SHR genomic data generated for this paper have accession number ERP001371. Reference BN data have accession number ERP000510. RNA-seq data are available via ArrayExpress accession E-MTAB-1029.

## Abbreviations

BN: Brown Norway; bp: base pair; CNV: copy number variant; FDR: false discovery rate; indel: insertion or deletion (in this paper <11 bp); LMP: long mate-pair; NGS: next generation sequencing; PCR: polymerase chain reaction; qPCR: quantitative PCR; QTL: quantitative trait locus; RI: recombinant inbred; SHR: spontaneously hypertensive rat; SNV: single nucleotide variant; SV: structural variant; UTR: untranslated region.

## Competing interests

The authors declare that they have no competing interests.

## Authors' contributions

MS produced BN-*Lx *DNA NGS libraries, performed analyses of RNA sequencing data and combinatorial analyses of genomic and RNA sequencing data and drafted the manuscript. SA performed SNV and indel analyses and participated in drafting the manuscript. SL produced RNA sequencing libraries. VG performed primary analyses of CNVs and SVs. FR performed mapping of sequencing data and participated in SNV and indel calling. LG carried out the SHR HiSeq 2000 sequencing. NL participated in NGS library production and sequencing. EB participated in NGS sequencing. SH performed qPCR experiments. SJ participated in generating the genomic SHR NGS libraries. MP participated in generating the tissue samples. TA participated in the design of the study and in drafting the manuscript. EC participated in the design of the study and in drafting the manuscript. All authors have read and approved the manuscript for publication.

## Supplementary Material

Additional file 1 **Summary of genomic sequencing data**. A table listing the genomic sequencing data. Click here for file

Additional file 2 **SNVs in SHR**. List of all SNVs found in the SHR strain. Click here for file

Additional file 3 **SNVs in BN-*Lx*. **List of all SNVs found in the BN-*Lx *strain. Click here for file

Additional file 4 **Validation of SNVs**. A table containing the details on the validated SNVs. Click here for file

Additional file 5 **Supplementary figures S1, S2 and S3**. Click here for file

Additional file 6 **Indels in SHR**. List of all indels found in the SHR strain. Click here for file

Additional file 7 **Indels in BN-*Lx*. **List of all indels found in the BN-*Lx *strain. Click here for file

Additional file 8 **Validation of indels**. A table that contains the details on the validated indels. Click here for file

Additional file 9 **Structural variants**. A list of all the SVs found in SHR and BN-*Lx*. Click here for file

Additional file 10 **Splice-site differences between BN-*Lx *and SHR**. A table listing splice sites that were found to be differentially used in SHR versus BN-*Lx*. Click here for file

Additional file 11 **Genes with stop codon and splice-site SNVs**. A table listing genes that contain SNVs in stop codons or essential splice sites. Click here for file

Additional file 12 **Genes with stop codon or frameshift indels**. A table listing genes that contain indels that are located in stop codons or that cause frameshifts. Click here for file

Additional file 13 **Genes with SVs**. A table listing genes that overlap SVs. Click here for file

Additional file 14 **Validation of stop codon SNVs**. A table that contains the details on the validation of the SNVs that are predicted to create new stop codons. Click here for file
