# Supplementary material for: Genetic basis of transcriptome differences between the founder strains of the rat HXB/BXH recombinant inbred panel
Source: Genome Biol. 2012 Apr 27;13(4):r31. doi: 10.1186/gb-2012-13-4-r31 (PMC3446305; doi:10.1186/gb-2012-13-4-r31)
Supplement: Additional file 1 — Summary of genomic sequencing data. A table listing the genomic sequencing data. [file gb-2012-13-4-r31-S1.PDF]

Tabel S1 overview of the genome sequencing data

	type	# libraries	mapped (Gbp)	median coverage
BN-Lx	fragment	2	32.7	
	paired-end	1	33.9	
	mate-pair	1	8.7	
			75.3	32X
SHR	fragment	2	23.4	
	paired-end	1	35.5	
	mate-pair	1	2	
			60.9	23X
