# Supplementary material for: Genetic basis of transcriptome differences between the founder strains of the rat HXB/BXH recombinant inbred panel
Source: Genome Biol. 2012 Apr 27;13(4):r31. doi: 10.1186/gb-2012-13-4-r31 (PMC3446305; doi:10.1186/gb-2012-13-4-r31)

Supplementary figure S1. Non-random distribution of SNVs in the BN-Lx genome.
SNVs in BN-Lx are clustered on the genome and found back in a subset of BN substrains. A. Number of SNVs per Mbp was determined across the genome and plotted per chromosome. Distinct peaks are seen demonstrating the SNVs are not randomly distributed. B. Heatmap of the genotypes of 12 BN substrains analyzed by the STAR consortium. Each row represents one SNV found in BN-Lx. Blue indicates reference alleles, yellow BN-Lx alleles and black are undetermined. Clustering shows that there is a group of BN-Lx specific SNVs. These are located in the peak on chromosome 8. BN-Lx is congenic for this region. The other SNVs separate the BN-substrains in two groups. These SNV regions likely reflect the BN substrain type that was used to create BN-Lx.

A

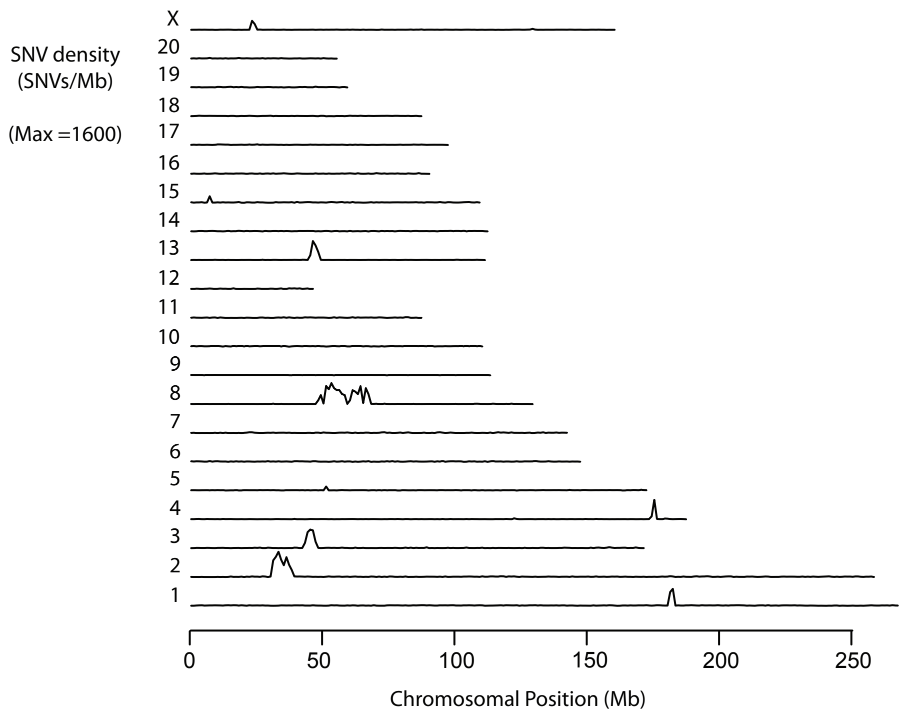

B

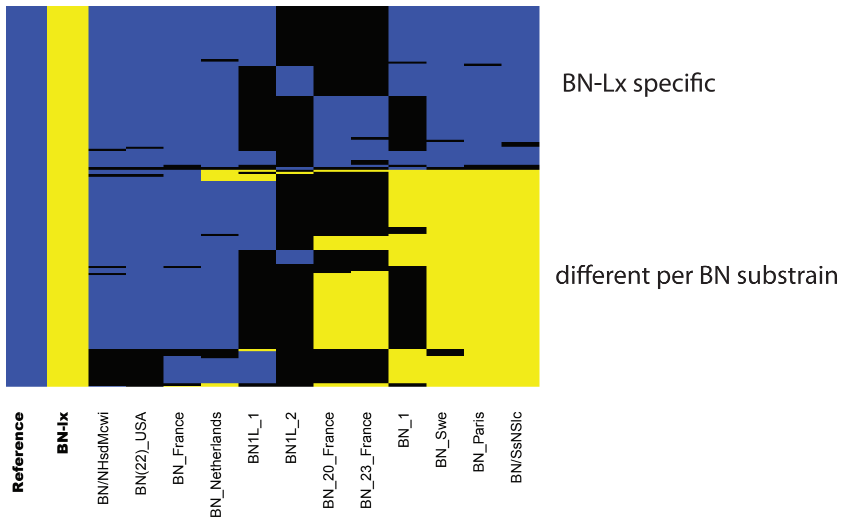

Figure S2. The duplicated gene MX2 shows increased expression level and transcripts from two alleles. A. genomic coverage (averaged over 999 base pairs) in SHR at the Mx2 locus . Increased coverage at the site of duplication is indicated with the white bar. B. expression levels as measured by normalized RNA-seq read counts in the coding regions of the MX2 gene. Asterisk indicates significant difference (FDR<0.05). C. Counts in RNA-seq reads of the different alleles found in the genomic sequencing in SHR.

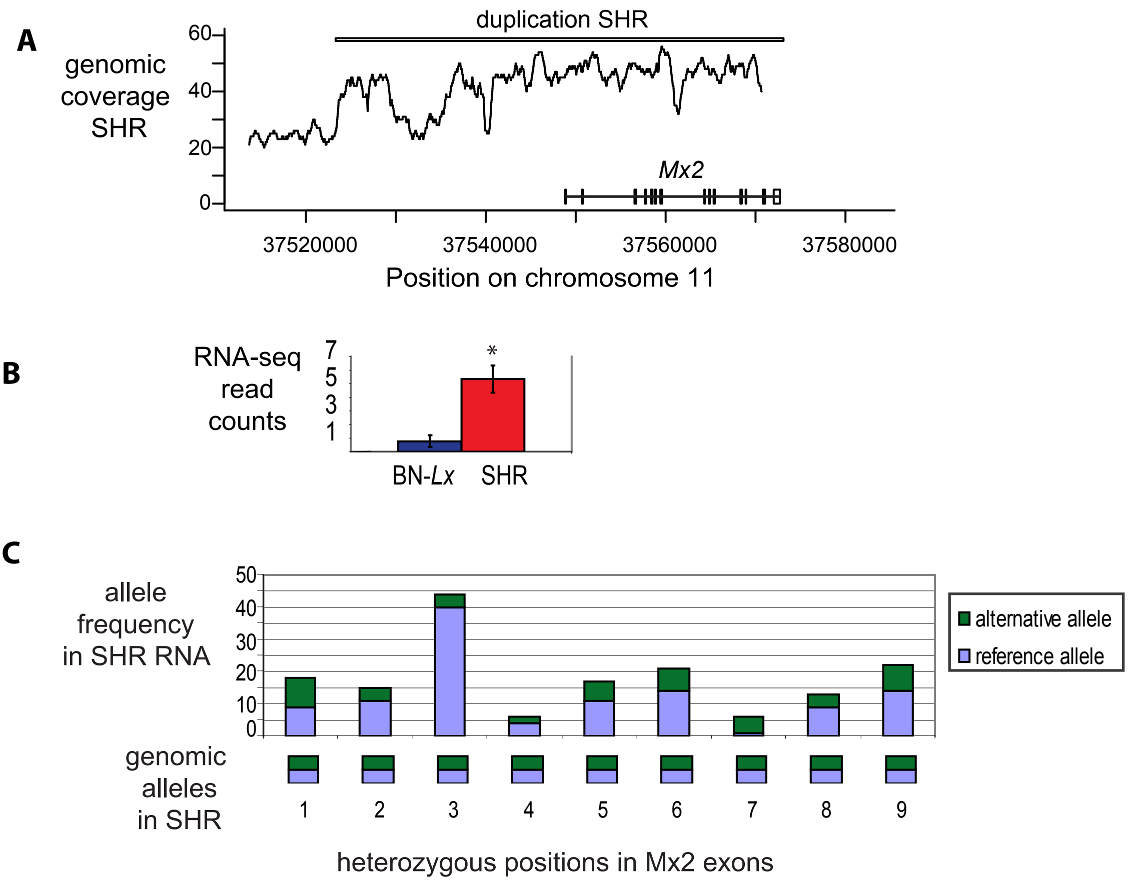

Figure S3. A SNV in an intron of the Fibrinogen Alpha gene in SHR adds 9 bases (3 amino acids) to the RNA produced for this gene in SHR. Cappillary sequencing reads on the cDNA are shown for both BN-Lx (top) and SHR (bottom).

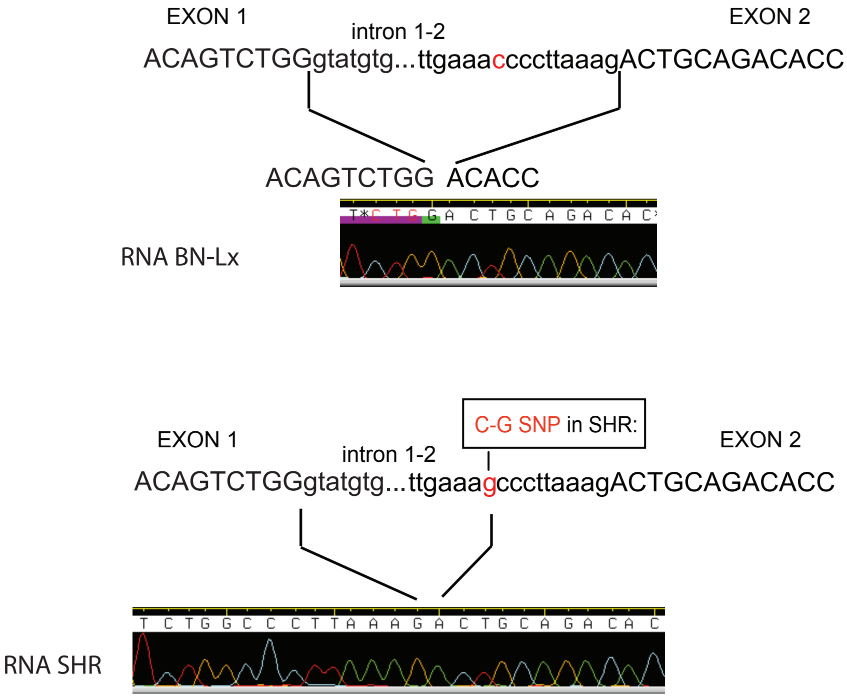

Supplement: Additional file 5 — Supplementary figures S1, S2 and S3. [file gb-2012-13-4-r31-S5.PDF]
