# Supplementary material for: Genetic basis of transcriptome differences between the founder strains of the rat HXB/BXH recombinant inbred panel
Source: Genome Biol. 2012 Apr 27;13(4):r31. doi: 10.1186/gb-2012-13-4-r31 (PMC3446305; doi:10.1186/gb-2012-13-4-r31)
Supplement: Additional file 10 — Splice-site differences between BN-Lx and SHR. A table listing splice sites that were found to be differentially used in SHR versus BN-Lx. [file gb-2012-13-4-r31-S10.PDF]

Table S2. List of splice junctions that are differential between BN-Lx and SHR. The number of reads mapped in each strain to the specific junction is shown and the ratio of reads mapping to the junction over total reads mapping to the coding part of the gene.

gene	junction	BN-Lx	SHR/OlaIpcv	BN-Lx junction/gene	SHR/OlaIpcv junction/gene
ENSRNOG00000020562	ENSRNOE00000200494 ENSRNOE00000200520	0	119	5.0E-04	6.6E-02
ENSRNOG00000033386	ENSRNOE00000171800 ENSRNOE00000171943	0	85	1.9E-04	1.5E-02
ENSRNOG00000011330	ENSRNOE00000107932 ENSRNOE00000254920	0	50	8.3E-04	3.0E-02
ENSRNOG00000025074	ENSRNOE00000238824 ENSRNOE00000238493	4	160	1.3E-03	4.3E-02
ENSRNOG00000017689	ENSRNOE00000170229 ENSRNOE00000170299	0	29	1.7E-04	5.2E-03
ENSRNOG00000033680	ENSRNOE00000279476 ENSRNOE00000202627	1	9	1.4E-03	3.8E-02
ENSRNOG00000012940	ENSRNOE00000125855 ENSRNOE00000125300	0	27	2.3E-03	5.5E-02
ENSRNOG00000015317	ENSRNOE00000295406 ENSRNOE00000307343	1	107	2.9E-03	6.5E-02
ENSRNOG00000020602	ENSRNOE00000331216 ENSRNOE00000356860	3	70	6.1E-03	1.2E-01
ENSRNOG00000006280	ENSRNOE00000058793 ENSRNOE00000058855	0	29	4.8E-04	8.1E-03
ENSRNOG00000015317	ENSRNOE00000295406 ENSRNOE00000320773	1	78	2.9E-03	4.7E-02
ENSRNOG00000014870	ENSRNOE00000142622 ENSRNOE00000142674	0	4	3.5E-03	5.6E-02
ENSRNOG00000003742	ENSRNOE00000035579 ENSRNOE00000035258	9	0	9.4E-02	5.8E-03
ENSRNOG00000017941	ENSRNOE00000323216 ENSRNOE00000295533	12	0	8.1E-03	4.9E-04
ENSRNOG00000030182	ENSRNOE00000312118 ENSRNOE00000361255	15	0	6.3E-03	3.8E-04
ENSRNOG00000026754	ENSRNOE00000159396 ENSRNOE00000159488	11	0	1.0E-02	5.9E-04
ENSRNOG00000012255	ENSRNOE00000275739 ENSRNOE00000275726	10	0	3.3E-02	1.9E-03
ENSRNOG00000006709	ENSRNOE00000062948 ENSRNOE00000066003	16	0	1.8E-03	1.1E-04
ENSRNOG00000023334	ENSRNOE00000021738 ENSRNOE00000323412	6	0	3.5E-02	2.0E-03
ENSRNOG00000029061	ENSRNOE00000281887 ENSRNOE00000277546	9	0	5.6E-02	3.2E-03
ENSRNOG00000015288	ENSRNOE00000147706 ENSRNOE00000260524	8	0	5.5E-02	3.1E-03
ENSRNOG00000004685	ENSRNOE00000292254 ENSRNOE00000044788	11	0	7.3E-02	4.1E-03
ENSRNOG00000018630	ENSRNOE00000357702 ENSRNOE00000181109	32	1	3.1E-02	1.7E-03
ENSRNOG00000004789	ENSRNOE00000045446 ENSRNOE00000045476	8	0	8.3E-02	4.5E-03
ENSRNOG00000013076	ENSRNOE00000125845 ENSRNOE00000126244	17	1	1.2E-01	6.5E-03
ENSRNOG00000011752	ENSRNOE00000111422 ENSRNOE00000111541	13	0	2.2E-02	1.2E-03
ENSRNOG00000025680	ENSRNOE00000306094 ENSRNOE00000333052	11	0	1.9E-02	9.7E-04
ENSRNOG00000007564	ENSRNOE00000072336 ENSRNOE00000072548	4	0	3.0E-02	1.5E-03
ENSRNOG00000005906	ENSRNOE00000308564 ENSRNOE00000230984	17	0	1.3E-01	6.0E-03
ENSRNOG00000000852	ENSRNOE00000007101 ENSRNOE00000007102	16	0	6.1E-03	2.8E-04
ENSRNOG00000018204	ENSRNOE00000175512 ENSRNOE00000175596	7	0	1.6E-02	7.1E-04
ENSRNOG00000017223	ENSRNOE00000165683 ENSRNOE00000166533	21	0	3.9E-03	1.8E-04
ENSRNOG00000005599	ENSRNOE00000239860 ENSRNOE00000285666	798	13	1.3E-01	2.2E-03
ENSRNOG00000020562	ENSRNOE00000200501 ENSRNOE00000200520	84	0	4.2E-02	5.5E-04
ENSRNOG00000038999	ENSRNOE00000303048 ENSRNOE00000364019	94	0	4.0E-02	4.8E-04
ENSRNOG00000028896	ENSRNOE00000337744 ENSRNOE00000379059	27	0	3.0E-02	2.9E-04
ENSRNOG00000024848	ENSRNOE00000238509 ENSRNOE00000238288	157	0	2.5E-02	1.6E-04
ENSRNOG00000024848	ENSRNOE00000238509 ENSRNOE00000238493	3330	11	5.2E-01	1.9E-03
